# Genetic and environmental influences on sleep-wake behaviors in adolescence

**DOI:** 10.1093/sleepadvances/zpab018

**Published:** 2021-10-22

**Authors:** Victoria S O’Callaghan, Narelle K Hansell, Wei Guo, Joanne S Carpenter, Haochang Shou, Lachlan T Strike, Jacob J Crouse, Kerrie McAloney, Katie L McMahon, Enda M Byrne, Jane M Burns, Nicholas G Martin, Ian B Hickie, Kathleen R Merikangas, Margaret J Wright

**Affiliations:** 1 Queensland Brain Institute, University of Queensland, Brisbane, Australia; 2 Genetic Epidemiology Branch, National Institute of Mental Health, National Institutes of Health Bethesda, MD, USA; 3 Brain and Mind Centre, University of Sydney, Sydney, Australia; 4 Department of Biostatistics, Epidemiology, and Informatics, University of Pennsylvania Perelman School of Medicine, Philadelphia, PA, USA; 5 QIMR Berghofer Medical Research Institute, Brisbane, Australia; 6 Queensland University of Technology, Brisbane, Australia; 7 Institute of Molecular Bioscience, University of Queensland, Brisbane, Australia; 8 Child Health Research Centre, University of Queensland, Brisbane, Australia; 9 Young and Well Cooperative Research Centre, Melbourne, Australia; 10 Centre for Advanced Imaging, University of Queensland, Brisbane, Australia

**Keywords:** sleep, adolescence, heritability, twins, genetics, actigraphy

## Abstract

**Study Objectives:**

To investigate the influence of genetic and environmental factors on sleep-wake behaviors across adolescence.

**Methods:**

Four hundred and ninety-five participants (aged 9–17; 55% females), including 93 monozygotic and 117 dizygotic twin pairs, and 75 unmatched twins, wore an accelerometry device and completed a sleep diary for 2 weeks.

**Results:**

Individual differences in sleep onset, wake time, and sleep midpoint were influenced by both additive genetic (44%–50% of total variance) and shared environmental (31%–42%) factors, with a predominant genetic influence for sleep duration (62%) and restorative sleep (43%). When stratified into younger (aged 9–14) and older (aged 16–17) subsamples, genetic sources were more prominent in older adolescents. The moderate correlation between sleep duration and midpoint (*r*P = −.43, *r*G = .54) was attributable to a common genetic source. Sleep-wake behaviors on school and nonschool nights were correlated (*r*P = .44–.72) and influenced by the same genetic and unique environmental factors. Genetic sources specific to night-type were also identified, for all behaviors except restorative sleep.

**Conclusions:**

There were strong genetic influences on sleep-wake phenotypes, particularly on sleep timing, in adolescence. Moreover, there may be common genetic influences underlying both sleep and circadian rhythms. The differences in sleep-wake behaviors on school and nonschool nights could be attributable to genetic factors involved in reactivity to environmental context.

**Statement of Significance**

We investigate the heritability of accelerometry measured sleep-wake behaviors in a large community sample of adolescents. Genetic factors play a primary role in sleep-wake behaviors. In younger (9–14) compared to older (16–17) adolescents, shared environment also contributes to sleep timing. Sleep duration and sleep midpoint were largely associated because of common genetic sources, suggesting sleep duration may be dictated by the same genes influencing circadian rhythms. Sources influencing sleep-wake behaviors on school and nonschool nights partially overlapped. Differences were due to distinct genetic sources, indicating the importance of distinguishing between night-type. Future work should extend the assessment of sleep-wake cycles to fully capture how these influences change across time, and to better understand the etiology of each trait.

## Introduction

Adolescence is a critical period with changes in multiple domains related to health and well-being. Changes in sleep across adolescence have been central to subsequent health and mental health in particular [1]. Changes in sleep patterns due to biological and societal factors have been repeatedly shown to produce the “perfect storm” that leads to enduring patterns of inadequate sleep [2]. In adults, many studies show that genetics strongly influence variation between individuals in sleep patterns [3–12]. However, adolescent twin studies have previously focused on sleep duration, and primarily used subjective rather than objective measures, such as accelerometry [13–15], with little known about genetic influences on sleep timing phenotypes that contribute to duration: sleep onset and sleep offset (wake time). These components are crucial to explore, as during adolescence, a shift occurs in the natural sleep-wake cycle, otherwise known as circadian rhythms [16, 17]. Beginning around the onset of puberty, a phase delay occurs in the biological clock of humans, resulting in a natural urge to go to sleep later and awaken later in the day. In fact, a study in Australian adolescents aged 9–18 found that with every year of age, sleep onset and wake time was delayed by up to 17 and 10 min, respectively, and sleep duration was decreased by up to 12 min [18].

Two studies [19, 20] have investigated the heritability of accelerometry measured 24-hour sleep-wake behaviors in adolescent twins. These devices provide estimates of sleep-wake behavior, circadian rhythms, and physical activity in an ecologically valid manner [21]. While for objective sleep, polysomnography (PSG) is the gold standard, accelerometry is psychometrically comparable and allows for more long-term measurement [22–24]. In the first study, Sletten et al. [19] investigated the heritability (*h*^2^; the proportion of phenotypic variance due to genetics [25]) of sleep-wake behaviors. The 25 monozygotic (MZ) and 41 dizygotic (DZ) twin pairs aged 12 wore an “Actiwatch-64” activity monitoring device (Respironics Inc, Bend, OR) for 2 weeks. Individual differences in sleep timing were influenced by additive genetic variation (*h*^2^ = 28% for sleep onset; 15% for wake time) but also by shared (family) environmental sources (65% and 76%, respectively). Other sleep phenotypes, including sleep duration, sleep onset latency, wake after sleep onset, sleep efficiency, and sleep fragmentation, were more strongly influenced by genetics (52%–81%), with little to no influence of shared environment. However, for establishing the importance of genetic and environmental factors, the sample size was modest, as shown by wide confidence intervals and noted by the authors.

The second study, by Inderkum and Tarokh [20], included 16 MZ and 10 DZ twin pairs with a mean age of 12. This study assessed sleep-wake patterns for a period of 6-months using “Jawbone UP” activity monitoring devices. The authors found lower heritability for school (*h*^2^ ranged between 0% and 38%) compared to nonschool nights (*h*^2^ ranged between 65% and 85%) for several sleep-wake phenotypes, including sleep onset, wake time, sleep duration, and time in bed, with shared environment accounting for more variation in school nights. This pattern was also found for sleep midpoint (school nights *h*^2^ = 14%, nonschool nights *h*^2^ = 90%), the time halfway between sleep onset and wake time. Sleep midpoint (or midsleep) can be used as a measure of circadian rhythms/chronotype, [26–28] with a later midpoint indicative of greater “eveningness” than “morningness” (colloquially known as being a night owl as opposed to a lark). However, heritability for sleep onset latency, wake after sleep onset, and sleep efficiency remained similar across night-types. While Inderkum and Tarokh [20], did not use bivariate analyses to explore why heritability may vary between night-type, they suggested that the observed heritability differences were due to increased shared environmental influence on school nights.

In contrast to the few objective studies of sleep-wake behaviors/patterns, several large twin studies have investigated subjectively rated sleep characteristics, primarily sleep duration. A recent meta-analysis of 23 studies across a wide age span (aged 6–88 years) yielded a heritability estimate of 28% for subjective sleep duration [29]. The heritability of usual sleep duration was shown to be age-dependent in a study of over 6000 adolescent twins [15], with a substantial increase in heritability between the ages of 12 (*h*^2^ = approximately 35%) and 20 (*h*^2^ = approximately 40%) and concomitant decrease in the effects of shared environmental influence in the older group. For other sleep-wake measures, it is also likely that shared environmental variance decreases across adolescence, as is seen in many other traits [30, 31].

Non-restorative sleep, defined as a subjective feeling of tiredness or not feeling refreshed upon waking despite a normal sleep duration [32] is another under-studied, yet important component of sleep, particularly in adolescents. Nearly 40% of adolescents in a large population study (*N* = 78 843) report that they “Never” or “Rarely” feel refreshed upon waking [33]. Furthermore, non-restorative sleep is associated with substantial cognitive and physical deficits [34]. By definition, non-restorative sleep occurs despite adequate time in bed and can indicate other underlying disorders such as insomnia [35], psychiatric problems [32, 36], or suicidal ideation [33]. Although no twin studies have investigated this trait in adolescents, a study of over 4000 adult twin pairs (mean age = 43.9) found a heritability of 37% for non-restorative sleep (i.e. waking up sleepy/tired [12]), whilst another in 2825 twin pairs from the Vietnam Era Twin Study of Ageing found a heritability of 21% [37].

Thus, while prior work shows genetic factors influence adolescent sleep-wake patterns, objective studies of 24-hour sleep-wake behaviors are limited by both sample size and age range and have yet to investigate whether the sources of variance change across adolescence. Additionally, one of the most central questions to our understanding of developmental influences on sleep is whether genetic factors underlie the phenotypic association between sleep duration and chronotype (*r*s = −.14 to −.34) particularly the reduced sleep duration among evening-type adolescents [38–41]. There is therefore an urgent need to explore sources of covariation and provide a better understanding of the etiology of adolescent sleep-wake behavior. Here, in a large adolescent twin study of sleep-wake behaviors, we estimate the influence of genetic and environmental factors, focusing on five key measures: sleep onset, wake time, sleep midpoint, sleep duration, and restorative sleep. To maximize the sample, we pool data from two Brisbane-based cohorts, who underwent identical measurement protocols, with similar family backgrounds. That is, families were predominantly of European ancestry and indicators such as parental education and occupation show that both samples reflect a broad cross-section of the south-east Queensland community. For the first time in an adolescent twin cohort, we investigate whether sources of variance in sleep-wake measures change across adolescence, disentangle the extent to which sleep duration and sleep midpoint are influenced by the same and/or different genetic sources, and explore whether the proportion of variance influenced by genetic sources (i.e. heritability) differs for sleep-wake measures on school and nonschool nights (e.g. does the influence of shared [family] environment reduce the heritability of sleep-wake measures on school nights?).

## Methods

### Participants

We used accelerometry data from two community adolescent twin cohorts: the Brisbane Adolescent Twin Study (BATS [42–44]) and the Queensland Twin Adolescent Brain (QTAB) project (Supplementary Table S1 includes a detailed description of these cohorts). The BATS cohort (57.65% female) included 55 identical (MZ) and 81 nonidentical (DZ) twin pairs, and 35 unpaired twins aged 12.06–17.70 years, with accelerometry data acquired between February 2014 and March 2018 (Note: Data from the BATS cohort used in Sletten et al. [19] was not incorporated here due to incompatibility issues between the accelerometry devices, see Supplementary Table S1). The QTAB cohort included 38 MZ and 36 DZ twin pairs and 40 unpaired twins aged 8.98–14.08 years (51.60% female), with data collected from June 2017 to October 2019. The combined sample consisted of 495 participants (55.35% females; aged 8.98–17.70): 93 MZ (50 MZ female [MZF]; 43 MZ male [MZM]) and 117 DZ (50 DZ female [DZF], 27 DZ male [DZM], and 40 DZ opposite sex [DZOS]) twin pairs, and 75 singletons (unpaired twins). For each cohort, both participants and their parent/guardian gave informed consent. Ethics approval was obtained from the relevant Human Research Ethics Committees. Participant honoraria varied across studies and covered multiple components. BATS participants received a movie pass or gift card/voucher to a maximum value of $30, and QTAB participants received $50.

### Procedure

Participants wore a wrist-mounted accelerometry recording device (GeneActiv, Activinsights, Kimbolton, UK) for 2 weeks. Accelerometry devices detect motion and can be used to estimate sleep from decreased movement. The device was worn day and night and only removed for showering/bathing/whilst around water or participating in contact sports. Every morning, participants completed a sleep diary, providing information on bedtimes, wake times, and restorative sleep [19]. Participants also recorded whether it was a school or nonschool night (i.e. a night preceding a weekend or holiday) for analyses involving night-type differences. The devices were set to record at a measurement frequency of 30 Hz per epoch. Participants wore the device, which was color coded to prevent mix-ups within pairs, on their nondominant wrist. On day 15, the devices were returned via postal service and the data downloaded using GENEActiv software.

### Measures

Raw accelerometry data were processed using the open source (and widely used [45]) R package GGIR (version 2.0 [46]). As outlined previously [47], GGIR was developed to process multi-day raw accelerometer data for physical activity and objective measures of sleep-wake behaviors [46]. With wrist-worn accelerometry, arm angle can be approximated in relation to the horizontal plane [48]. Using this information, GGIR classifies inactivity periods (potential sleep periods) as consecutive 5 s epochs where the angle of an individual’s arm does not change more than five degrees in relation to the horizontal plane for 5 min [48]. Inactivity periods during the self-reported sleep window (recorded in participant’s sleep diaries) were classified as sleep periods. Sleep measures extracted using this algorithm have been shown to be comparable to PSG [46, 48].

Post-processing was completed using the package postGGIR [49], see https://cran.r-project.org/web/packages/postGGIR/index.html. We removed any nights with extreme (± 6 h) disparities between the sleep diary and accelerometry data (e.g. a bedtime noted as 8 pm but an onset recorded as 5 am), nights with missing data (e.g. due to non-wear time or device malfunction), as well as participants with less than seven nights of data across the 14-night study period (*n* = 48). As is standard in the field [19, 48, 50, 51], we extracted estimates of sleep onset time (the start of the first sustained nocturnal inactivity period during the sleep window), wake time (the end of the last sustained inactivity period), and sleep duration (the sum of estimated sleep periods), and calculated sleep midpoint as the time halfway between onset and wake [26, 50, 52] to use as a measure of circadian rhythm. To be able to measure chronotype with accelerometry, sleep must be measured on nonschool/free days without the use of an alarm clock and with a correction for accumulated sleep debt [28, 53–55]. As this information was unavailable, we could not classify individuals into morning/evening types but instead report their circadian behavior, as has been done previously [20, 27, 28]. We also included a measure of restorative sleep from the sleep diary, where participants answered, “When I woke up this morning, I felt” and indicated either “Refreshed” or “Tired.” Responses were coded as “Refreshed” = 1 or “Tired” = 0 then averaged across all days to identify the percentage of nights of restorative sleep for the testing period. For each sleep-wake measure, we computed the average across the total number of nights (7–14 nights) and separately for school and nonschool nights, with the latter defined as nights preceding weekends, public and school holidays. For wake time, school days were counted as Monday to Fridays. On average, participants had a total of 12.10 nights of actigraphy data overall (range = 7–14), as well as 5.23 school nights (range = 0–10; since participation for 46 participants occurred entirely during school holidays), and 6.87 nonschool nights (range = 2–13). This was in keeping with the recommended five to seven nights required for reliable estimates of sleep-wake behaviors [56].

We assessed pubertal status using the Pubertal Development Scale [57, 58], categorizing participants into five stages (1—prepubertal, 2—early pubertal, 3—midpubertal, 4 – late pubertal, and 5 – postpubertal) —see Supplementary Table S1 for cohort demographics. These categories are based on secondary sexual characteristics, including skin changes, growth spurts, and body hair growth for both males and females, as well as change in voice and facial hair for males and breast development and menarche for females. Each participant’s height and weight were measured by a research assistant (60%) or self-reported (40%) to the nearest millimeter for height and gram for weight (Supplementary Table S1). As only a few families were asked whether the twins shared a bedroom, we could not adjust for any variance due to this. However, when tested on a subset (33 MZ, 27 DZ pairs) we found no evidence that shared room status had an effect (*p* > .626 for all phenotypes). Furthermore, others have shown that intrapair (co-twin) differences for sleep-wake measures do not differ based on shared room status [19, 20].

### Statistical analysis

We scaled the sleep-wake phenotypes into *z*-scores, winsorizing outliers (*z* > ± 3.29) and removing extreme outliers (z > ± 4.00). There was a total of 24 outliers across four phenotypes: sleep onset (five winsorized, one removed), wake time (five winsorized), sleep midpoint (four winsorized, three removed), and sleep duration (four winsorized, two removed); restorative sleep did not contain any outliers. We conducted model-fitting analyses, estimated maximum likelihood twin correlations, and subsequently phenotypic, genetic, and environmental correlations using OpenMx 2.17.2 [59] in R version 3.6.2. [60] Saturated models, where all parameters are estimated freely, were fitted to the data. Preliminary analyses showed that for all variables except sleep midpoint, we could equate means and variance of MZ and DZ twins, and no differences were found based on birth order or zygosity. Although we found a scalar sex limitation [61] for sleep midpoint, given our modest sample size we collapsed across sex for genetic modeling (i.e. we used two zygosity groups [MZ and DZ] rather than five). We then examined fixed effects of age, sex, age × sex, age [2], height, weight, and puberty. As age and puberty correlated highly (*r* = .73), we examined the effects of puberty before and after regressing the effects of age, and separately for females and males. We found no cohort effects. All genetic analyses were run using residuals after regressing out the effects of age and sex.

Identical (MZ) twins develop from the splitting of a single zygote and share virtually 100% of their genes, whereas nonidentical (DZ) twins develop from two individual zygotes and share on average 50% of their genes. However, twins brought up in the same household are exposed to the same family environment growing up. Therefore, when MZ twins are more similar in a particular trait than DZ twins, we can infer genetic influence. This comparison is the basis for twin studies. Here, we used a classic twin design [62, 63], whereby we partitioned variance in a trait into additive genetics (A), shared or common environmental (C) (i.e. sources that influence both twins such as parenting style), and non-shared (unique) environmental (E) sources (i.e. sources that influence one twin but not the other such as one twin being bullied). Variance due to non-shared environmental sources also includes measurement error. In twin models, within twin pair correlations between additive genetic factors are fixed at MZ*r* = 1.0 and DZ*r* = 0.5 (based on sharing of genetic material). Within twin pair correlations between shared or common environmental factors are fixed at 1.0 for both MZ and DZ pairs (as these factors influences both twins, regardless of whether twins are identical or not). Finally, correlations between non-shared (unique) environmental factors are fixed at 0 for both MZ and DZ pairs (as these factors are specific to one twin, regardless of whether they are identical or not).

Note, whilst additive genetic variance represents the total average effect of all alleles that are influencing a trait, nonadditive genetic variance (D) can also be estimated and represents genetic dominance (allelic interactions at the same loci) [64]. However, we cannot estimate the influence of dominance (D) as well as shared environment (C) within the same model using the current design (the sample would have to include twins raised apart as well as twin raised together). Given the significant shared environmental influence found in previous studies [19, 20], we chose to not investigate dominance effects. Overall, the twin correlations supported an ACE model.

**Figure 1.**
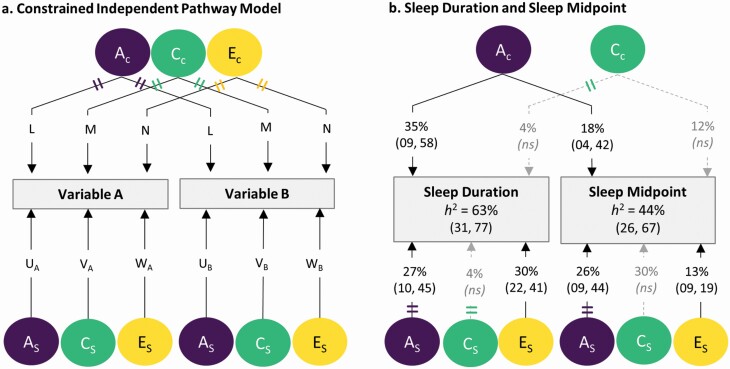
(a) Path diagram for the constrained bivariate independent pathway model. A_c_ = common additive genetic source, C_c_ = common shared environmental source, E_c_ = common unique environmental source, A_s_ = specific additive genetic influence, C_s_ = specific shared environmental influence, E_s_ = specific unique environmental influence. L, M, N = A_c_ C_c_ and E_c_ parameter estimates. In order to be identified, this model requires constraints to reduce the number of parameter estimates. One possibility, as shown here, is to constrain the paths from A_c_ (as well as C_c_ and E_c_) to variables A and B to be equal. The choice of constraints is guided by the magnitude of free parameter estimates observed in an unconstrained model. Matching colored double dashes indicate paths constrained to be equal. U_A_, V_A_, W_A_, and U_B_, V_B_, W_B_ represent parameter estimates of specific A, C, and E influences on variable A and B. Squares indicate observed variables and circles indicate latent variables. (b) Independent pathway model showing the association between sleep duration and sleep midpoint is entirely due to a common genetic source, that is, 56% (i.e. 35/63) of the genetic variance in sleep duration and 41% (18/44) of the genetic variance in sleep midpoint overlapped. Parameter estimates (95% confidence intervals) are shown as a percentage of total variance. Some non-significant estimates are large, for example, 30% of the variance in sleep midpoint was due to a specific shared (family) environmental source (C_s_), with 42% of the variance attributed to shared environmental sources (C_s_ + C_c_) and matching that reported in Table 3. *h*^2^ = heritability estimate (i.e. percentage of total variance due to genetic sources). Nonsignificant paths are shown as dashed lines (those with zero influence are not shown).

When only one trait is examined, this is known as a univariate twin model. However, we can also estimate the proportion of covariance between two traits that is due to additive genetics (A), shared or common environmental (C), and non-shared (unique) environmental (E) sources. This is known as a bivariate (or multivariate) twin model. Bivariate models, with more sources of information, provide greater power than univariate models to determine model estimates [64]. Whilst there are a number of different bivariate models that can be used, here we focused on two equivalent approaches [65]: the common factor independent pathway model and the Cholesky decomposition. The common factor independent pathway model (also known as an independent pathway model) further proportions sources of variance into those shared (common) between two traits/variables and those that are specific (example of model shown in Figure 1A). Whilst the Cholesky decomposition can also provide this information, it must be run twice with variable order reversed (for further explanation see Supplementary Figure S1).

We first took a univariate approach to estimate the variance in sleep-wake behaviors, using data for all available nights and across all ages (i.e. the full sample). As there was a strong effect of age (Supplementary Table S2), we stratified the sample and estimated sources of variance for younger (aged 9–14) and older (aged 16–17) subsamples, using residuals specific to each. For each of the sleep-wake phenotypes, to find the most parsimonious model without compromising model fit, full ACE path models (i.e. allowing for additive genetic [A], shared or common environment [C], and non-shared [unique] environment [E]) were compared to sub-models with individual components constrained (i.e. allowing for AE, CE, or only E sources of influence). We compared model fit using Akaike’s information criteria (AIC), where a lower AIC value represented a better model fit.

Then, using data collected across all nights and over all ages we used bivariate modeling to investigate sources of shared variance between sleep duration and sleep midpoint, which has not yet been explored in adolescent twins. We compared estimates from an independent pathway model to those from a Cholesky decomposition [65], which has identical overall goodness of fit [65]. The Cholesky decomposition was run twice, in order to estimate overlapping and specific influences for both variables, with estimates shown in the supplementary material as a conceptual model for clarity (see Supplementary Figure S1). Paths with zero or close to zero influence were subsequently dropped from analyses. We then used the same bivariate approach to investigate sources of variance common and specific to night-type (school and nonschool) for each of the sleep-wake phenotypes.

## Results

The means and standard deviations for the five sleep-wake phenotypes for the full sample across all nights of participation, as well as for school and nonschool nights and younger and older subsamples, are shown in Table 1. Sleep timing measures were delayed on nonschool nights compared with school nights: sleep onset was delayed by 25 min, wake time by 15 min, and sleep midpoint by 20 min. There was also a higher frequency of restorative sleep for nonschool (46% ± 32%) compared to school nights (40% ± 32%). In contrast, sleep duration was similar for school and nonschool nights, with an average of 7 h and 36 min (± 00:36) each night. When we stratified the sample by age, older adolescents (16–17 years) had a significantly later sleep onset, wake time, and sleep midpoint, shorter sleep duration, and a decrease in the frequency of restorative sleep across all nights compared to younger adolescents (9–14 years, Table 1).

**Table 1.** Means ± standard deviation [range; *N*] for sleep phenotypes averaged across 2 weeks (all nights), as well as school and nonschool nights, across all ages (*N* = 495; 93 MZ and 117 DZ pairs), and all nights of participation for the younger (*N* = 313; 58 MZ and 69 DZ pairs) and older (*N* = 182; 35 MZ and 48 DZ pairs) subsamples

	Full sample: Aged 9–17 years				Younger subsample Aged 9–14 years (All nights)	Older subsample Aged 16–17 years (All nights)	
Phenotype	All nights	School nights	Nonschool nights†	P			P
Sleep onset (hh:mm)	21:51 ± 01:00 [19:35–01:11; 495]	21:38 ± 01:00 [19:51–24:45; 448]	22:03 ± 01:04 [19:30–01:35; 495]	1.92e-09*	21:27 ± 00:52 [19:35–00:33; 313]	22.32 ± 00:51 [20:55-01:11; 182]	<2.20e-16*
Wake time (hh:mm)	06:46 ± 00:42 [04:43–09:07; 495]	06:39 ± 00:39 [04:41–09:02; 453]	06:54 ± 00:49 [04:10–09:22; 495]	4.10e-07*	06:38 ± 00:42 [04:43–09:05; 313]	07:03 ± 00:38 [05:25-09:07; 182]	1.67e-10*
Sleep midpoint (hh:mm)	02:19 ± 00:47 [00:20–05:04; 495]	02:10 ± 00:46 [00:19–04:55; 447]	02:29 ± 00:51 [00:20–05:08; 494]	3.47e-16*	02:03 ± 00:44 [00:20–04:10; 313]	02:38 ± 00:37 [01:21-05:04; 182]	<2.20e-16*
Sleep duration (hh:mm)	07:36 ± 00:36 [05:47–09:15; 495]	07:35 ± 00:42 [05:27–09:24; 448]	07:37 ± 01:07 [03:33–10:39; 478]	.487	7:46 ± 00:33 [05:36–09:15; 313]	7:18 ± 00:37 [05:47-09:07; 182]	7.50e-16*
Restorative sleep‡ (%)	44% ± 28% [0%–100%; 493]	40% ± 32% [0–100%; 456]	46% ± 32% [0%-100%; 493]	.00814*	47% ± 28% [0%-100%; 311]	37% ± 29% [0%-100%; 182]	.000305*

^†^Nonschool nights refer to nights preceding weekends, school holidays and public holidays; nonschool nights include 46 participants where data was collected entirely during school holidays.

^‡^Obtained from sleep diary: mean percentage of nights of restorative sleep (e.g. on average participants had restorative sleep on 40% of all school nights), all other sleep variables taken from actigraphy.

*Significant difference between school and nonschool nights for the full sample, and between younger and older adolescents for all nights of participation, p < .01 (.05/5); no significant differences in means between MZ (monozygotic) and DZ (dizygotic) twin pairs.

In the full sample, we found that for every year increase in age, sleep onset, wake time, and sleep midpoint were delayed by 14 min (i.e. 0.24 h), 6 min, and 10 min respectively, and that sleep duration decreased by 7 min per year and was 15 min shorter for males than females (Supplementary Table S2). These findings held for both school and nonschool nights. For restorative sleep, there was a 3% decrease with each year of age on nonschool nights but no significant change with age for school nights. Using age and sex corrected residuals, we found no evidence for the effects of age × sex, age [2], height, or weight. Further, pubertal status was not significant for either females or males after age correction (Supplementary Table S2).

Table 2 shows the maximum likelihood MZ and DZ twin correlations (see Supplementary Table S3 for the twin correlations for the five zygosity groups). The correlation coefficients for all five sleep-wake measures for MZ twin pairs were larger than for DZ twin pairs, suggesting a genetic influence (e.g. MZ*r* ranged .49–.87 and DZ*r* ranged .03–.65 for measures examined across all nights). For sleep timing measures, and younger adolescents in particular, the DZ twin correlations were greater than half the MZ correlations, suggesting some of the variance was due to shared environmental influence. Means and variances for all sleep-wake phenotypes were not significantly different between MZ and DZ twins. There were also no differences in age, height, weight, and pubertal status between zygosity type.

**Table 2:** Maximum likelihood monozygotic (MZ) and dizygotic (DZ) twin pair correlations (with 95% confidence intervals) for sleep phenotypes for the full sample for all nights of participation, school, and nonschool nights, and for all nights of participation for younger and older subsamples

	Full sample [93 MZ/117 DZ pairs]						Younger subsample [58 MZ/69 DZ pairs] (All nights)		Older subsample [35 MZ/48 DZ pairs] (All nights)	
	All nights		School nights		Nonschool nights†					
Phenotype	MZ	DZ	MZ	DZ	MZ	DZ	MZ	DZ	MZ	DZ
Sleep onset	.87 (.81, .90)	.61 (.50, .70)	.72 (.60, .80)	.40 (.24, .53)	.86 (.80, .90)	.60 (.48, .69)	.93 (.90, .96)	.71 (.59, .80)	.76 (.57, .86)	.44 (.16, .64)
Wake time	.78 (.69, .84)	.56 (.42, .67)	.76 (.65, .83)	.51 (.36, .62)	.75 (.65, .82)	.47 (.30, .59)	.81 (.71, 0.87)	.73 (.61, .81)	.70 (.47, .83)	.19 (−.13, .46)
Sleep midpoint	.87 (.81, .91)	.65 (.53, .73)	.79 (.69, .85)	.50 (.36, .62)	.83 (.76, .88)	.62 (.50, .71)	.92 (.88, .95)	.77 (.66, .84)	.74 (.54, .85)	.37 (.07, .60)
Sleep duration	.70 (.59, 0.78)	.39 (.23, .52)	.62 (.48, .73)	.34 (.17, .48)	.74 (.64, .81)	.27 (.10, .43)	.69 (.55, .79)	.43 (.23, .58)	.72 (.53, .83)	.30 (−.01, .54)
Restorative Sleep‡	.49 (.34, .61)	.03 (−.17, .23)	.47 (.30, .60)	.14 (−.06, .32)	.30 (.12, .45)	−.21 (−.39, 0)	0.57 (.39, .70)	.02 (−.23, .27)	.40 (.08, .62)	.03 (−.27, .33)

^†^Non-school (nights) precede weekends, school holidays and public holidays.

^‡^Obtained from sleep diary; Standardised residuals of age and sex used; full sample age range = 9 to 18 years; Younger subsample age range = 9 to 14 years; Older subsample age range = 16 to 17 years.

**Figure 2.**
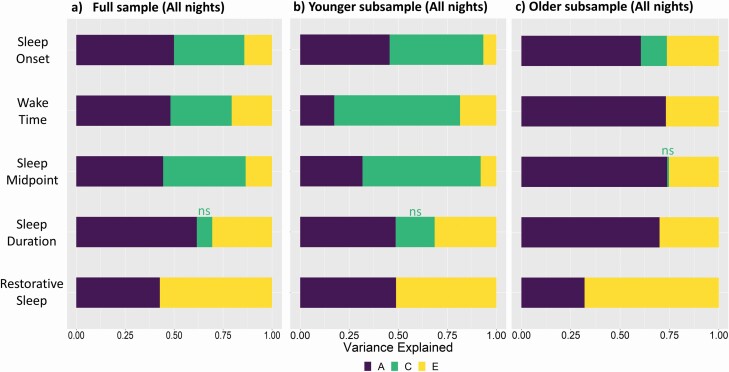
Proportion of variance in sleep-wake phenotypes across all nights due to additive genetic (A), shared environment (C), and unique environment (E) for the (a) full sample (across all ages) (b) younger and (c) older adolescents. There is a notable shift in the sources of variance for younger compared to older adolescents: in younger adolescents (aged 9–14 years) there is a substantial shared (family) environmental influence on sleep onset, wake time, and sleep midpoint, but little or no influence of shared environment for older adolescents (aged 16–17 years), so that the proportion of genetic variance is increased. This is not seen in sleep duration and restorative sleep where the influence of shared (family) environment is non-significant for both age groups. Variances are derived from the ACE Model (Table 3). Nonsignificant estimates are shown as “ns”.

Heritability estimates (*h*^2^) from the full sample ACE models ranged from a low of 0.43 for restorative sleep (i.e. genetic influence accounted for 43% of total variance) to a high of 0.62 for sleep duration. Across adolescence, as shown in Figure 2, there was a change in the relative proportions of genetic and environmental variance, such that shared environmental influences (e.g. family environment) identified in the full sample for sleep onset, wake time, and sleep midpoint appear to be driven by shared environmental influences in younger adolescents, with little or no indication for this source influencing older adolescents. Thus, in older adolescents with the reduction in shared environmental variance, genetic sources accounted for more of the total variance (Sleep Onset: Younger = 46% (95% CI = 29 to 70), Older = 60% (57 to 84); Wake Time: Younger = 17% (1 to 43), Older = 73% (54 to 84); Sleep Midpoint: Younger = 32% (17 to 53), Older = 74% (23 to 85)). This pattern was not found for sleep duration or restorative sleep, where even in young adolescents the importance of shared environmental factors was not significant. Table 3 shows the results of model-fitting analyses. In the full sample, and both younger and older adolescents, an AE model, allowing for only additive genetic (A) and unique environmental (E) influences, provided the best fitting model for sleep duration and restorative sleep, and for older adolescents only, for sleep onset, wake time, and sleep midpoint.

**Table 3.** Model parameters and proportion of variance in sleep phenotypes due to additive genetic (A), common (shared) environment (C), and unique (non-shared) environment (E) for the best fitting models

Phenotype	Sample	Model	AIC	A	C	E
Sleep onset	Full	**ACE**	**254.14**	**0.50 (0.31, 0.74)**	**0.36 (0.13, 0.54)**	**0.14 (0.10, 0.20)**
		AE	260.31	0.86 (0.81, 0.90)	–	0.14 (0.10, 0.19)
		CE	276.75	–	0.71 (0.64, 0.77)	0.29 (0.23, 0.36)
	Younger	**ACE**	**106.03**	**0.46 (0.29, 0.70)**	**0.48 (0.46, 0.65)**	**0.07 (0.04, 0.10)**
		AE	115.72	0.93 (0.90, 0.95)	–	0.07 (0.05, 0.10)
		CE	135.04	–	0.80 (0.73, 0.85)	0.20 (0.15, 0.27)
	Older	ACE	127.06	0.60 (0.11, 0.84)	0.13 (0.00, 0.53)	0.26 (0.16, 0.45)
		**AE**	**125.32**	**0.74 (0.57, 0.84)**	**–**	**0.26 (0.16, 0.43)**
		CE	130.58	–	0.57 (0.39, 0.70)	0.43 (0.30, 0.61)
Wake time	Full	**ACE**	**291.79**	**0.48 (0.24, 0.77)**	**0.31 (0.04, 0.53)**	**0.21 (0.15, 0.29)**
		AE	294.53	0.80 (0.73, 0.85)	–	0.20 (0.15, 0.27)
		CE	304.45	–	0.66 (0.58, 0.73)	0.34 (0.27, 0.42)
	Younger	**ACE**	**157.50**	**0.17 (0.01, 0.43)**	**0.64 (0.40, 0.81)**	**0.18 (0.12, 0.28)**
		AE	172.35	0.82 (0.75, 0.88)	–	0.18 (0.12, 0.25)
		CE	157.77	–	0.77 (0.69, 0.83)	0.23 (0.17, 0.31)
	Older	ACE	131.10	0.73 (0.54, 0.84)	0.00 (0.00, 0.26)	0.27 (0.16, 0.46)
		**AE**	**129.10**	**0.73 (0.54, 0.84)**	**–**	**0.27 (0.16, 0.46)**
		CE	140.5	–	0.46 (0.27, 0.62)	0.54 (0.38, 0.73)
Sleep midpoint	Full	**ACE**	**245.31**	**0.44 (0.26, 0.67)**	**0.42 (0.20, 0.59)**	**0.13 (0.10, 0.19)**
		AE	254.55	0.87 (0.82, 0.90)	–	0.13 (0.10, 0.18)
		CE	265.83	–	0.74 (0.67, 0.79)	0.26 (0.21, 0.33)
	Younger	**ACE**	**108.05**	**0.32 (0.17, 0.53)**	**0.60 (0.39, 0.74)**	**0.08 (0.05, 0.12)**
		AE	125.26	0.92 (0.88, 0.94)	–	0.08 (0.06, 0.12)
		CE	123.45	–	0.83 (0.77, 0.87)	0.17 (0.13, 0.23)
	Older	ACE	124.48	0.74 (0.23, 0.85)	0.01 (0.00, 0.45)	0.25 (0.15, 0.43)
		**AE**	**122.48**	**0.75 (0.58, 0.85)**	**–**	**0.25 (0.15, 0.42)**
		CE	130.07	–	0.55 (0.46, 0.68)	0.45 (0.32, 0.54)
Sleep duration	Full	ACE	336.05	0.62 (0.29, 0.77)	0.08 (0.00, 0.35)	0.31 (0.23, 0.41)
		**AE**	**334.30**	**0.70 (0.60, 0.77)**	**–**	**0.30 (0.23, 0.40)**
		CE	347.46	–	0.52 (0.42, 0.61)	0.48 (0.39, 0.58)
	Younger	ACE	213.25	0.49 (0.09, 0.78)	0.20 (0.00, 0.52)	0.31 (0.22, 0.46)
		**AE**	**212.33**	**0.70 (0.57, 0.79)**	**–**	**0.30 (0.21, 0.43)**
		CE	216.85			
	Older	ACE	131.55	0.70 (0.24, 0.82)	0.00 (0.00, 0.38)	0.30 (0.18, 0.49)
		**AE**	**129.55**	**0.70 (0.51, 0.82)**	–	**0.30 (0.18, 0.49)**
		CE	137.08	–	0.49 (0.30, 0.64)	0.51 (0.36, 0.70)
Restorative sleep†	Full	ACE	396.41	0.43 (0.19, 0.56)	0.00 (0.00, 0.17)	0.57 (0.44, 0.73)
		**AE**	**394.41**	**0.43 (0.27, 0.56)**	**–**	**0.57 (0.44, 0.73)**
		CE	402.77	–	0.27 (0.14, 0.39)	0.73 (0.61, 0.86)
	Younger	ACE	247.75	0.49 (0.19, 0.64)	0.00 (0.00, 0.21)	0.51 (0.36, 0.70)
		**AE**	**245.75**	**0.49 (0.30, 0.64)**	**–**	**0.51 (0.36, 0.70)**
		CE	253.26	–	0.31 (0.15, 0.45)	0.69 (0.55, 0.85)
	Older	ACE	155.76	0.32 (0.00, 0.55)	0.00 (0.00, 0.37)	0.68 (0.45, 0.97)
		**AE**	**153.76**	**0.32 (0.03, 0.55)**	**–**	**0.68 (0.45, 0.97)**
		CE	155.07	–	0.21 (0.00, 0.41)	0.79 (0.59, 1.00)

Bold texts and values indicate the best fitting model; note, to facilitate comparison across phenotypes ACE estimates are reported in the text.

Full sample age range = 9 to 18 years, 93 MZ and 117 DZ pairs; Younger subsample age range = 9 to 14 years, 58 MZ pairs, 69 DZ pairs; Older subsample age range = 16 to 17 years, 35 MZ pairs, 48 DZ pairs; Standardised residuals of age (separately for each group) and sex used.

^†^Taken from sleep diary.

As expected, sleep onset and wake time were correlated (phenotypic *r* (*r*P) = .71), and both measures correlated highly with sleep midpoint (*r*Ps > .88). Longer sleep duration was associated with earlier sleep onset (phenotypic *r* [*rP*] = −.62, genetic r (*r*G) = −.66) and sleep midpoint (*rP* = −.43; *r*G = −.54) but was not significantly correlated with wake time. Restorative sleep did not correlate with any accelerometry measures (see Supplementary Table S4 for phenotypic and genetic correlations among all variables).

The association between sleep duration and sleep midpoint (*r*P = −.43) was largely due to a common genetic source (genetic correlation *r*G = .54), with no significant influence from overlapping shared or unique environment, such that up to 56% (i.e. 0.35/0.63) of the genetic variance in sleep duration and 41% of the genetic variance in sleep midpoint overlapped (Figure 1B and Supplementary Figure S2). Essentially, we found the association between sleep duration and sleep midpoint to be stronger in identical twins (who share 100% of their genetic material) than nonidentical twins (who on average, share only 50%). While the independent pathway model apportioned more genetic variance to a common genetic source than the Cholesky decomposition, confidence intervals for estimates overlapped across models, and others using the same modeling approach have shown a similar pattern of results (see Figure 3 in Loehlin et al. [65]).

**Figure 3.**
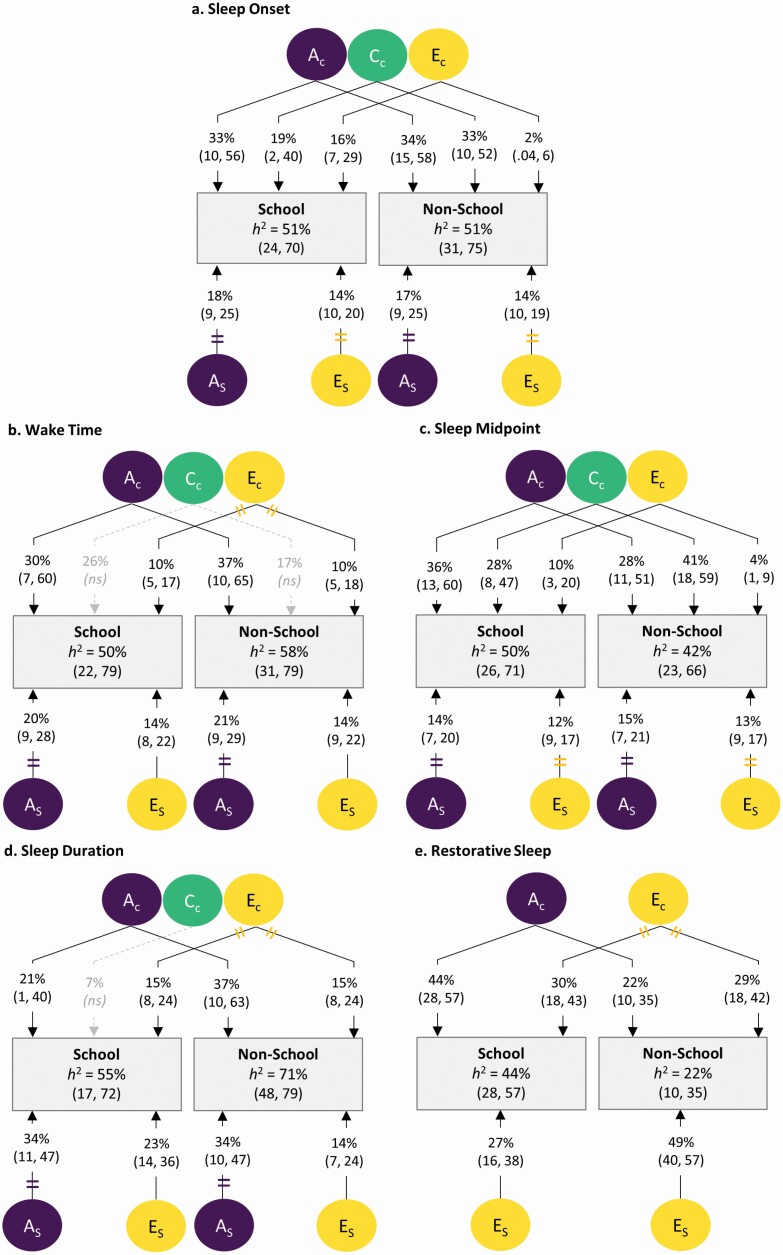
Sources of variance on school and nonschool nights for (a) sleep onset, (b) wake time, (c) sleep midpoint, (d) sleep duration, and (e) restorative sleep. Bivariate independent pathway models show that for all accelerometry measures (sleep onset, wake time, sleep midpoint, sleep duration) both common (A_c_) and specific (A_s_) genetic sources influence school and non-school nights in adolescence, with 36% to 71% heritability; In contrast, variance due to shared (family) environment (C_s_) completely overlapped, with no evidence of a shared environmental influence specific to night-type. For restorative sleep, genetic variance for school and non-school nights completely overlapped (A_c_), and there was no genetic variance specific to night-type, and no evidence of any variance due to shared (family) environment; *h*^2^ = heritability estimate (i.e., the percentage of variance influenced by genetic sources). Nonsignificant paths are shown as dashed lines and those with zero influence are not shown. Matching colored double dashes indicate paths constrained to be equal. Squares indicate observed variables and circles indicate latent variables; A_c_ = common additive genetic source, C_c_ = common shared environmental source, E_c_ = common unique environmental source, A_s_ = specific additive genetic influence, C_s_ = specific shared environmental influence, E_s_ = specific unique environmental influence.

Sleep-wake behaviors on school and nonschool nights were moderately (sleep duration: *rP* = .44) to highly correlated (restorative sleep: .60, sleep onset and wake time: .64, sleep midpoint: .72, Supplementary Table S4). A common additive genetic source (Ac) between sleep onset on school nights and sleep onset on nonschool nights were found. This additive genetic source accounted for just over a third of the total variance in sleep onset on both school (33%) and nonschool (34%) nights (Figure 3A, see Supplementary Figure S3a for Cholesky decomposition). A common shared (family) environmental source (Cc) between sleep onset on school nights and sleep onset on nonschool nights accounted for a further 19 and 33% of the variance in sleep onset on school and nonschool nights respectively. Remaining covariance was due to non-shared (unique) environmental sources (Ec) and accounted for 16% of variance in sleep onset on school nights and 2% on nonschool nights. These are due to environmental factors experienced by only one twin, and therefore are not correlated across twins. However, uncorrelated measurement error is also captured by this component. A similar pattern was observed for wake time (Figure 3B and Supplementary Figure S3B for Cholesky), although the overlapping shared environmental source was non-significant, and for sleep midpoint (Figures 3C and Supplementary Figure S3C). In contrast, for both sleep duration and restorative sleep (Figure 3D–E and Supplementary Figure S3D–E) covariation between school and nonschool nights were primarily due to the same genetic source, with no contribution due to shared environmental factors. Even so, with the exception of restorative sleep, for all four accelerometry measures, and in particular for sleep duration, some of the genetic variance was specific to night-type (i.e. specific A [As] ranged from 14% to 34%; Figure 3A–D), whereas all shared environmental variance overlapped for school and nonschool nights (Figure 3A–D and Supplementary Figure S3A–E).

Heritability estimates did not differ significantly for school and nonschool nights (Figure 3A–E and Supplementary Figure S3A–E) though we note that confidence intervals were wide. Similar estimates were found for sleep onset (51% for both school and nonschool), wake time (50% and 58%, respectively), and sleep midpoint (50% and 42%, respectively). There was a trend for lower heritability on school nights for sleep duration (school: 55% (17, 72 95% CIs); nonschool: 71% (48 to 79)) and a higher heritability for school (44% (28 to 57)) compared to nonschool nights (22% (10 to 35)) for restorative sleep.

## Discussion

In a large community sample of adolescents, aged 9–17, we estimated the genetic and environmental sources of variance in four accelerometry measures of sleep-wake behaviors, as well as a subjective measure of restorative sleep. Across a maximum of 14 nights, additive genetic sources accounted for 43%–62% of the variance in adolescent sleep-wake behaviors, with shared (family) environment accounting for between 0% and 42%. When we stratified the sample by age into younger (9–14 years) and older (16–17 years) groups, variance due to shared environment was no longer prominent in older adolescents, and sleep-wake behaviors were more strongly attributable to genetic sources. Across adolescence we showed that shorter sleep duration was associated with a later sleep midpoint. The novel finding that this association was largely due to common genetic factors accounting for approximately half of the genetic variance, demonstrated that there is some overlap in the genetic etiology of sleep duration and sleep midpoint. Further, while we found heritability was somewhat similar for school and nonschool nights, sleep-wake behaviors on school nights were only moderately correlated with those of nonschool nights.

Our finding that up to half of the variance in adolescent sleep-wake behavior is due to a genetic source is in line with previous studies [19, 20], though our heritability estimates are slightly higher likely due to our larger age range (i.e. the inclusion of adolescents up to age 17). Heritability was most substantial for sleep duration, with up to 62% of variance due to genetic factors, and in general agreement with both objective [19, 20] and self-report [15, 29] measures of sleep duration. Compared with sleep timing measures, there was very little influence of shared environment on duration, consistent with previous findings [3–7, 13, 66]. This suggests that although parents may dictate bedtimes and rise times, genetic factors contribute more than environmental factors to sleep duration in adolescents. These findings also highlight the now universal finding of suboptimal sleep duration across adolescence [67], with a nearly 30-minute lower mean sleep duration than the minimum recommended for this age group (8 to 10 h) [68]. Mean sleep onset, wake time, and sleep midpoint, as well as 44% of nights with restorative sleep [33], was also consistent with previous studies [19, 20].

Our results followed the expected pattern of sleep timing becoming later and duration becoming shorter in adolescents with increased age [18, 69, 70]. Sleep onset and wake time were both delayed by 14 and 6 min per year respectively, and sleep duration decreased by approximately 7 min per year. These changes are in line with findings from a similar cohort [18]. We also found that males slept approximately 15 mins less than females, consistent with previous findings [18]. After correcting for age, we did not find a significant effect of puberty on any of the sleep-wake phenotypes, thus, it may be that cross-sectional analyses are not adequate to detect such effects. Indeed, Sadeh et al. [71] found sleep-wake behaviors (sleep onset, sleep duration, sleep efficiency, number of awakenings) in 9- to 11-year-olds at Time 1 did not predict puberty cross-sectionally but did predict puberty longitudinally at Time 2 (approximately 1 year later), after partialling out the effects of age and sex.

Across all four of the accelerometry measured sleep-wake phenotypes, heritability was generally lower in younger (9 to 14 years) compared with older (16 to 17 years) adolescents. This was largely due to the prominence of a shared environmental source influencing younger adolescents, most notable for the sleep timing phenotypes of sleep onset, wake time, and sleep midpoint. Earlier studies in 12-year-old twins have indicated a minor role of genetic factors for sleep timing phenotypes, with a more considerable influence of shared environment [19, 20]. This increased shared environmental influence in younger adolescents could be due to parental decisions, family schedules, sport/social activities, etc., which have been shown to influence bedtimes and rise times [19, 20, 72] but no longer have a significant effect on older adolescents. This is seen in many other traits [30, 31] where decreasing shared environmental influences, as individuals become more independent, lead to higher heritability estimates. However, this does not necessarily imply that genetic sources account for more variance with age, but rather a larger proportion of variance with age. So, if environmental variance becomes less influential and total variance is reduced, and if genetic influence remains unchanged, then the consequence is increased heritability (i.e. genes now account for a greater proportion of the total variance). Studies in older adolescents [73] (aged 17) and adults [8–11], found no shared environmental influence on self-reported chronotype. Likewise, here, we show that in older adolescents, shared environment no longer influences sleep midpoint, albeit using a different circadian measure.

In line with previous studies [38–41], we found an association between sleep duration and sleep midpoint (*r*P = −.43) such that later midpoint was associated with shorter sleep duration. This reinforces the importance of the mismatch between greater eveningness and socially driven school start time that leads to sleep deprivation [69, 74, 75]. Despite the role of social structure driving this disparity, we found that genetic factors underlie both sleep midpoint and duration. That is, the association between sleep duration and sleep midpoint was stronger for identical twins than it was for non-identical twins, indicating that genes are influencing the association. Social demands, on the other hand, could be expected to influence identical and non-identical twins equally and would be identified as a shared environmental influence. While we did see some indication of a shared environmental influence, these estimates were not significant, and larger samples will be required to better understand all sources of influence. A recent genome-wide association (GWAS) study (*N* = 446 118) estimated a genetic correlation of *r*G = −.10 between self-reported sleep duration and chronotype [76] and identified a genetic variant (rs9382445) associated with sleep duration that was previously associated with chronotype [77]. Whilst in the present study we cannot confirm the exact genes that sleep duration and sleep midpoint have in common, we speculate that genes involved in circadian rhythm sleep-wake disorders, such as delayed sleep phase syndrome, may be involved [78–80]. Future studies are needed to discover new genetic variants common and unique to both to elucidate other biological pathways.

As expected, for all sleep-wake behaviors there was a moderately strong association between school and nonschool nights, which for wake time, sleep duration, and restorative sleep was largely due to a common genetic source. In addition, except for restorative sleep, our bivariate analyses indicated that night-type differences were due to distinct genetic sources, with remaining variance due to environmental factors unique to the individual. This was particularly evident for sleep duration where up to half of the genetic variance was specific to night-type, suggesting the influence of different factors or heritable traits on each night-type (school vs. nonschool), with school start time moderating sleep behaviors on school nights. While we can only speculate, the personality trait conscientiousness [81, 82] which is associated with sleep hygiene behaviors, including consistency in total sleep time [83], as well as differences in morningness/eveningness could play a role. Further, while we found that heritability estimates did not differ significantly for night-type, a trend for sleep duration having lower heritability on school nights was consistent with a prior study, likely due to a shared (family) environmental factor influencing school nights as speculated previously [20].

A phenotype which had not yet been investigated in adolescent twins was non-restorative sleep (the feeling of tiredness even after a normal sleep duration [32]). In contrast to the accelerometry sleep phenotypes, non-restorative sleep did not follow the same pattern of common variance across school and nonschool nights. Notably there was no shared environmental influence and no age differences for restorative sleep, and our heritability estimate was similar to that previously reported in adult twins [12, 37]. Thus, unlike sleep timing phenotypes, individual-specific genetic and environmental factors rather than shared environmental factors contribute to the quality of sleep or feeling refreshed after sleep in our adolescent sample. This is particularly interesting because this subjective appraisal of sleep quality did not correlate with any of the accelerometry measured sleep-wake behaviors. Additionally, non-restorative sleep has been more closely tied to mood disorders [32, 33, 36] than other sleep measures have, highlighting the necessity to investigate different sleep phenotypes (subjective and objective) in future studies, particularly if investigating the link between sleep and mental health.

This study contributes to our understanding of the genetic and environmental factors influencing accelerometry assessed sleep parameters in adolescence, a critical period for the development of social, cognitive, and physical milestones. Our findings regarding changes in the balance between environmental and genetic influences on sleep-wake behaviors across adolescence has not been previously documented. In addition, we used bivariate methods to directly compare sleep phenotypes collected on school and nonschool nights, and to compare sleep duration and sleep midpoint. Further, this is the largest sample of adolescent twins that enabled us to examine younger and older adolescence across this critical transition period using pooled data from the same population, twins living in South East Queensland, that was assessed using the same methods and protocols.

However, this study also has a number of limitations. Firstly, most participants (91%) had a maximum of four nights of data contributing to their nonschool night means, so that our estimates for nonschool nights may not be as precise as if we had collected data over a time frame longer than 14 nights. For instance, the lower heritability for restorative sleep on nonschool nights may have been from increased measurement error due to assessing fewer nonschool nights. Although participants overall had the recommended minimum of five to seven nights worth of data [56], future studies should consider testing larger samples over extended periods to obtain more accurate estimates of common and specific genetic variance for school and nonschool nights. In particular, sample size has limited our ability to detect shared environmental influences. As a reference, in univariate analyses, a sample size of approximately 1000 twin pairs is necessary to detect a shared environmental influence accounting for 15% of the variance (with 80% power, a type-1 error rate of .05 for a fixed value of the DZ correlation of .50) [84]. Further, our age effects are cross-sectional and thus limit conclusions that can be drawn. Efforts to increase sample size and to fully utilize longitudinal data by harmonizing data collected from different accelerometry devices (e.g. Actiwatch, GeneActiv) are underway (e.g. mMARCH.org).

## Conclusions

Not only do sleep-wake patterns change across adolescence, but so do the sources of variance, with shared environment becoming less important in later years. Different sources of variance influence sleep-wake phenotypes on school and nonschool nights suggesting that individual differences may not only be determined by sleep genes, but genes specific to traits that influence sleep-wake behavior. The strong genetic overlap between sleep duration and sleep midpoint requires future study to elucidate their biological pathways and better understand their etiologies.

## Supplementary Material

zpab018_suppl_Supplementary_Material
