# Supplementary material for: Genetic and environmental influences on sleep-wake behaviors in adolescence
Source: Sleep Adv. 2021 Oct 22;2(1):zpab018. doi: 10.1093/sleepadvances/zpab018 (PMC10104400; doi:10.1093/sleepadvances/zpab018)
Supplement: zpab018_suppl_Supplementary_Material [file zpab018_suppl_supplementary_material.pdf]

Supplementary Material

Genetic and Environmental Influences on Sleep-Wake Behaviours in Adolescence

Table of Contents

Tables	Title	Page Number
S1	Demographics for the combined sample, the Brisbane Adolescent Twin Study (BATS) and Queensland Twin Adolescent Brain (QTAB) project	2
S2	Effect of covariates on sleep phenotypes across all ages and all nights as well as school and non-school nights (puberty examined separately for females and males)	3
S3	Twin correlations (95%CI) for monozygotic female (MZF), monozygotic male (MZM), dizygotic female (DZF), dizygotic male (DZM) dizygotic opposite sex (DZOS) pairs	4
S4	Phenotypic (below diagonal) and genetic (above diagonal) correlations (95%CIs) between sleep phenotypes across all participation nights in the full sample and phenotypic correlations between school and non-school nights for all sleep phenotypes	5
Figures	Title	Page Number
S1 a-c	Example of a) a bivariate Cholesky decomposition, b) a reversed bivariate Cholesky decomposition, c) a conceptual model derived from a bivariate Cholesky decomposition	6
S2	Conceptual model derived from a Cholesky decompositions between sleep duration and sleep midpoint	8
S3 a-e	Conceptual model derived from a Cholesky decompositions between school and non-school nights for a) sleep onset, b) wake time, c) sleep midpoint, d) sleep duration, and e) restorative sleep	9

Table S1: Demographics for the combined sample, the Brisbane Adolescent Twin Study (BATS) and Queensland Twin Adolescent Brain (QTAB) project

	Combined Sample	BATS	QTAB
N	495	307	188
% Females	55.35	57.65	51.60
Mean Age (years) ± SD [Range]	13.60 ± 2.48 [8.98 – 17.70]	14.84 ± 2.09 [12.06 - 17.70]	11.46 ± 1.10 [8.98 – 14.08]
Median Age (Modal Age/s)	12.45 (12, 16)	16.31 (12, 16)	11.54 (11)
Puberty stage 1 (*n*) females / males	31 / 28	2 / 5	29 / 26
Puberty stage 2 (*n*) females / males	20 / 54	0 / 24	20 / 30
Puberty stage 3 (*n*) females / males	84 / 78	42 / 38	42 / 46
Puberty stage 4 (*n*) females / males	87 / 44	73 / 38	14 / 6
Puberty stage 5 (*n*) females / males	39 / 1	39 / 1	0 / 0
Height (cm)	156.18 ± 14.05 [120.20 - 196.00]	163.24 ± 11.67 [137.00 - 196.00]	145.23 ± 9.39 [120.20 - 168.20]
Weight (kg)	47.48 ± 15.01 [21.50 - 110.00]	53.51 ± 14.78 [28.40 - 110.00]	38.35 ± 8.95 [21.50 - 66.00]

BATS participants were a community sample residing in South East Queensland, Australia, recruited through primary and secondary schools, word of mouth, and the Australian Twin Registry (now known as Twins Research Australia); see Wright and Martin[42] for a description of the cohort and study components as at 2004. For both BATS and QTAB studies, families were excluded if either twin had a history of head injury or a major neurological/psychiatric disorder. Further exclusions were made in the QTAB sample due to MRI contra-indications (e.g., having braces), however, samples did not differ based on neuropsychiatric criteria. Data from the first 132 participants of BATS, collected using an Actiwatch-64 device (Respironics Inc, Bend, OR, USA), were analysed in the study of Sletten and colleagues,[19] however, these participants could not be included here due to incompatibility issues with GeneActiv devices. GeneActiv actigraphy data were available for a total of 372 participants in BATS. After data quality exclusions (e.g., insufficient data, malfunctions, outliers; *n* = 65), 307 participants from BATS remained, with 121 participants aged 12, four participants aged 14, and 182 aged 16 or 17. Zygosity for all same-sex twins was determined by genotyping. QTAB participants were recruited from the Queensland Twin (QTwin) registry at QIMR Berghofer, the Twins Research Australia (TRA) registry, or were an expression of interest via the QTAB project website; all participants resided in South East Queensland. Accelerometry data were available for 53% (*n* = 223 individuals) of the QTAB wave 1 sample (422 families), and after data quality exclusions (e.g., insufficient data, device malfunctions, outliers; *n* = 35), 188 QTAB participants remained. Zygosity for same-sex twins was determined by parental questionnaire (80%) or physical similarity (20%) and will be verified in the future with genotyping.

Table S2: Effect of covariates on sleep phenotypes across all ages and all nights as well as school and non-school[a] nights (puberty examined separately for females and males)

	Age		Sex [b]		Cohort		Age*Sex [c]		Age^{2} [c]		Height [c]		Weight [c]		Puberty (Females, *n* = 274) [d]		Puberty (Males, *n* = 221) [d]	
Phenotype	*B*	*p*	*B*	*p*	*B*	*p*	*B*	*p*	*B*	*p*	*B*	*p*	*B*	*p*	*B*	*p*	*B*	*p*
Sleep Onset																		
All nights	0.24	2.112E-18*	0.037	0.637	-0.048	0.715	-0.0050	0.873	0.00014	0.988	2.45E-06	0.995	2.45E-04	0.542	0.29	7.12E-07*	0.13	0.0279
School	0.26	2.616E-22*	-0.039	0.641	0.011	0.931	-0.023	0.494	-0.0065	0.502	3.06E-04	0.542	-3.12E-04	0.554	0.34	3.31E-06*	0.30	0.000377
Non-school	0.23	4.942E-14*	0.081	0.372	-0.21	0.163	0.0053	0.885	0.000797	0.942	-2.73E-04	0.561	6.73E-04	0.164	0.33	3.50E-07*	0.034	0.623
Wake Time *All nights*	0.093	8.890E-06*	-0.10	0.109	-0.038	0.718	0.034	0.185	0.00502	0.508	-1.16E-04	0.754	1.56E-04	0.685	0.14	0.00279	0.056	0.236
School	0.10	6.841E-07*	-0.16	0.0104	0.067	0.503	0.038	0.125	0.0038	0.603	1.25E-06	0.997	5.46E-05	0.884	0.13	0.00331	0.10	0.0628
Non-school	0.084	4.932E-04	-0.084	0.286	-0.0081	0.947	0.029	0.361	0.0048	0.589	-1.12E-04	0.814	6.26E-05	0.900	0.13	0.0198	0.057	0.370
Sleep Midpoint *All nights*	0.17	1.407E-13*	-0.016	0.798	-0.041	0.708	0.017	0.502	0.0029	0.716	-7.95E-05	0.804	2.32E-04	0.478	0.21	4.38E-06*	0.058	0.148
School	0.18	2.637E-18*	-0.12	0.0650	-0.011	0.912	-0.0046	0.858	-0.000601	0.936	1.69E-04	0.619	-1.42E-04	0.687	0.23	5.42E-06*	0.17	0.00203
Non-school	0.17	9.266E-11*	0.028	0.714	-0.15	0.243	0.021	0.492	0.0021	0.818	-2.63E-04	0.502	4.81E-04	0.234	0.21	3.23E-05*	0.018	0.711
Sleep Duration *All nights*	-0.11	1.144E-11*	-0.26	3.205E-06*	0.043	0.601	0.031	0.159	0.0060	0.315	3.83E-05	0.913	-1.32E-04	0.722	-0.22	9.64E-07*	-0.10	0.0423
School	-0.12	1.514E-10*	-0.26	5.558E-05*	-0.083	0.387	0.032	0.227	0.0079	0.270	-6.60E-05	0.878	8.48E-05	0.853	-0.26	6.52E-06*	-0.16	0.00915
Non-school	-0.18	2.453E-08*	-0.37	6.748E-04	-0.013	0.932	-0.043	0.321	0.0044	0.698	4.12E-04	0.571	-4.24E-04	0.583	-0.28	0.000967	-0.24	0.0232
Restorative Sleep (%) ^	-2.05	0.00575	4.30	0.113	0.40	0.905	-0.45	0.680	-0.095	0.730	-3.23E-02	0.129	2.97E-02	0.194	-1.90	0.319	-7.40	0.00957
All nights *School*	-1.50	0.0957	4.30	0.177	2.20	0.632	-0.57	0.659	-0.045	0.895	-2.27E-02	0.354	1.90E-02	0.469	-1.40	0.552	-7.10	0.0317
Non-school	-2.99	9.0795E-05*	4.40	0.132	1.40	0.724	-0.38	0.743	-0.14	0.614	-3.53E-02	0.168	3.25E-02	0.241	-5.70	0.00893	-9.80	0.00139*

*$p < .00037$ (.05/135)

[a] Non-school nights refer to those preceding weekends and holidays (public and school)

[b] Sex is coded as female = 0 and male = 1

[c] Age and sex corrected residuals of the sleep measures used for these analyses (i.e. to assess covariates Age*Sex, Age^2, Height, Weight)

[d] Significant puberty effects disappear when age and sex corrected residuals of the sleep measures are used. Puberty is coded as stage 1 = 1, stage 2 = 2…stage 5 = 5. The female sample comprised 50 MZF pairs, 50 DZF pairs, 74 singletons. The male sample comprised 43 MZM pairs, 27 DZM pairs, 81 singletons. Females on average were slightly shorter (means = 155.19cm ± 12.84 vs 157.41cm ± 15.38) and weighed less (means = 46.98kg ± 14.07 vs 48.09kg ± 16.14) than males, albeit these differences were not significant. Males were less far along in terms of puberty stages than females (see Table S1).

^from sleep diary

NOTE: Beta values for all significant covariates are expressed in hours (i.e. raw scores used for covariates and phenotypes)

Table S3: Twin correlations (95%CI) for monozygotic female (MZF), monozygotic male (MZM), dizygotic female (DZF), dizygotic male (DZM) dizygotic opposite sex (DZOS) pairs

Phenotype[&]	MZF (*n* = 50 pairs)	MZM (*n* = 43 pairs)	DZF (*n* = 50 pairs)	DZM (*n* = 27 pairs)	DZOS (*n* = 40 pairs)
Sleep Onset	.83 (.74, .89)	.91 (.85, .94)	.70 (.54, .80)	.66 (.42, .80)	.51 (.28, .66)
Wake Time	.71 (.56, .81)	.88 (.80, .92)	.65 (.46, .77)	.71 (.45, .83)	.39 (.13, .59)
Sleep Midpoint	.81 (.70, .87)	.95 (.91, .97)	.77 (.64, .85)	.74 (.52, .84)	.48 (.24, .64)
Sleep Duration	.70 (.56, .80)	.70 (.51, .81)	.18 (-.11, .42)	.48 (.17, .67)	.57 (.33, .72)
Restorative Sleep^	.51 (.28, .67)	.47 (.26, .63)	.12 (-.19, .40)	.36 (-.17, .64)	-.13 (-.39, .16)

[&]Standardised residuals of age and sex used; ^ from sleep diary.

NOTE: With the exception of Sleep Midpoint, MZF correlations could be set equal to MZM, DZF correlations could set equal to DZM, and DZ opposite-sex correlations could be set equal to the DZ same-sex correlation. Thus, there is no suggestion of differences in magnitude or source for males and females for Sleep Onset, Wake Time, Sleep Duration, and Restorative Sleep. However, as indicated by the 95% confidence intervals shown above, for Sleep Midpoint, the MZM correlation was higher than the MZF correlation. This suggests that genetic sources of influence may be stronger for males than females for Sleep Midpoint. Furthermore, assumption test H1c was significant for midpoint suggesting presence of scalar sex limitation ($p = .00023$) meaning $p < .00278$ (.05/180). Nonetheless, given our modest sample size, we have collapsed across sex (i.e. having one MZ and one DZ group) for genetic modelling.

Table S4: Phenotypic (below diagonal) and genetic (above diagonal) correlations (95%CIs) between sleep phenotypes across all participation nights in the full sample and phenotypic correlations between school and non-school nights for all sleep phenotypes

Phenotype[&]	Onset	Wake	Sleep Midpoint	Sleep Duration	Restorative Sleep	School and non-school *r*P
Sleep Onset	-	.50 (.20, .74)	.91 (.83, .96)	-.66 (-.86, -.41)	*r*P *ns*	.64 (.57, .69)
Wake Time	.71 (.65, .76)	-	.82 (.65, .91)	*r*P *ns*	*r*P *ns*	.64 (.58, .70)
Sleep Midpoint	.95 (.94, .96)	.89 (.87, .91)	-	-.54 (-.88, -.21)	*r*P *ns*	.72 (.66, .76)
Sleep Duration	-.62 (-.55, -.68)	*-.09 (-.19, .01)*	-.43 (-.35, -.51)	-	*r*P *ns*	.44 (.35, .52)
Restorative Sleep ^	*-.04 (-.13, .06)*	*-.06 (-.16, .03)*	*-.05 (-.14, .04)*	*.02 (-.08, .11)*	-	.60 (.54, .66)

[&]Standardised residuals of age and sex used; *r*P, phenotypic correlation; non-significant phenotypic correlations in italics; genetic correlations are shown for significant phenotypic associations; ^from sleep diary.

Figure S1

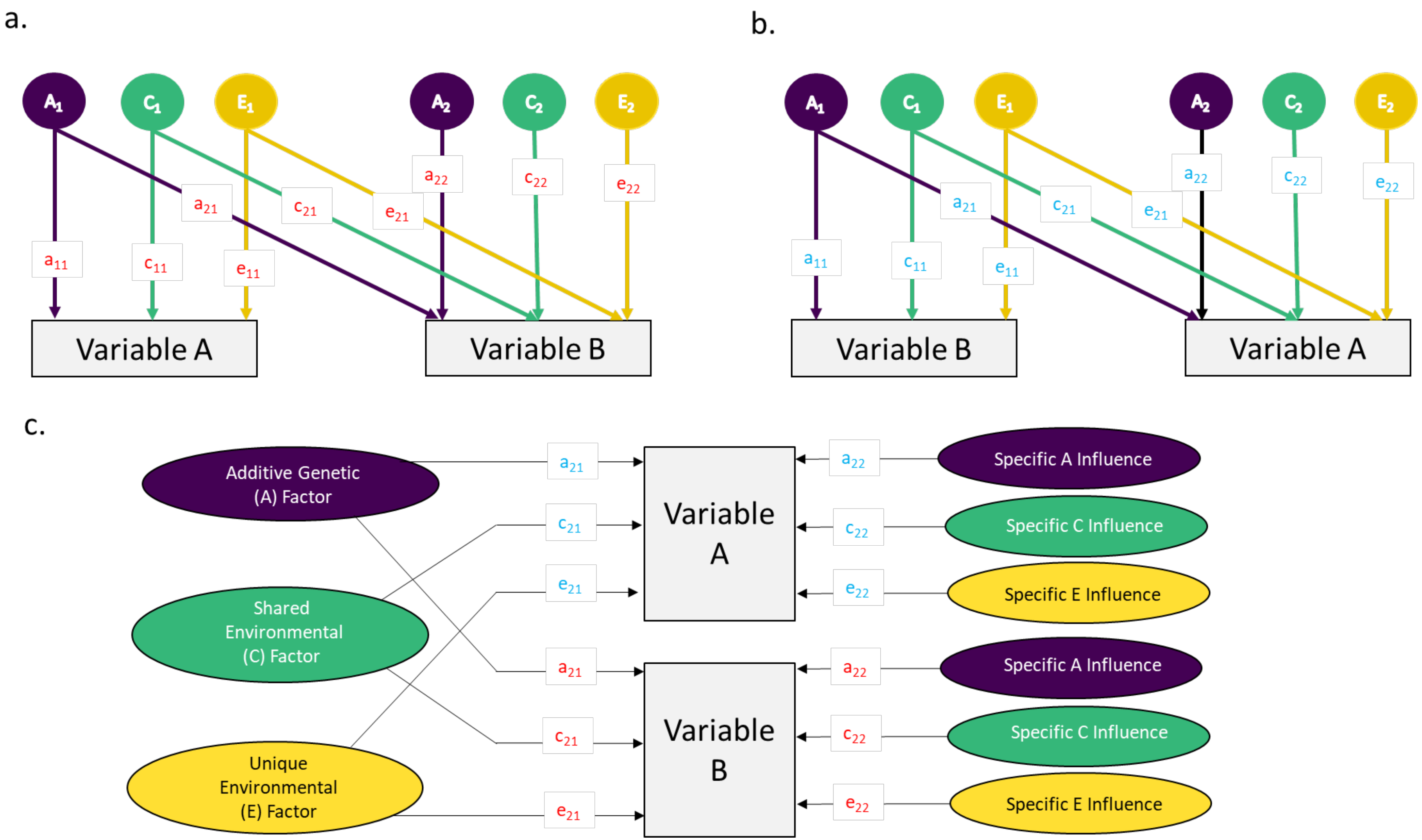

a.
A1
C1
E1
A2
C2
E2
a11
c11
e11
a21
c21
e21
a22
c22
e22
Variable A
Variable B
b.
A1
C1
E1
A2
C2
E2
a11
c11
e11
a21
c21
e21
a22
c22
e22
Variable B
Variable A
c.
Additive Genetic (A) Factor
Shared Environmental (C) Factor
Unique Environmental (E) Factor
a21
c21
e21
a21
c21
e21
Variable A
Variable B
a22
c22
e22
a22
c22
e22
Specific A Influence
Specific C Influence
Specific E Influence
Specific A Influence
Specific C Influence
Specific E Influence

Figure S1 a-c. Example of **a)** a bivariate Cholesky decomposition with variable A entered first and **b)** a reversed bivariate Cholesky decomposition with variable B entered first. This allows both shared and specific sources of variance to be estimated for each sleep phenotype; squares/rectangles indicate observed variables; circles indicate latent variables; A_1, C_1, E_1 represent genetic, shared environmental, and unique environmental sources influencing both Variable A and Variable B, whereas A_2, C_2, and E_2 represent genetic, shared environmental, and unique environmental sources influencing Variable B only; **c)** Example of a conceptual Cholesky decomposition with values a21, c21, e21, a22, c22, and e22 derived from S1b (blue) or S1a (red). h^2 = heritability estimate; dashed lines represent non-significant pathways

Figure S2

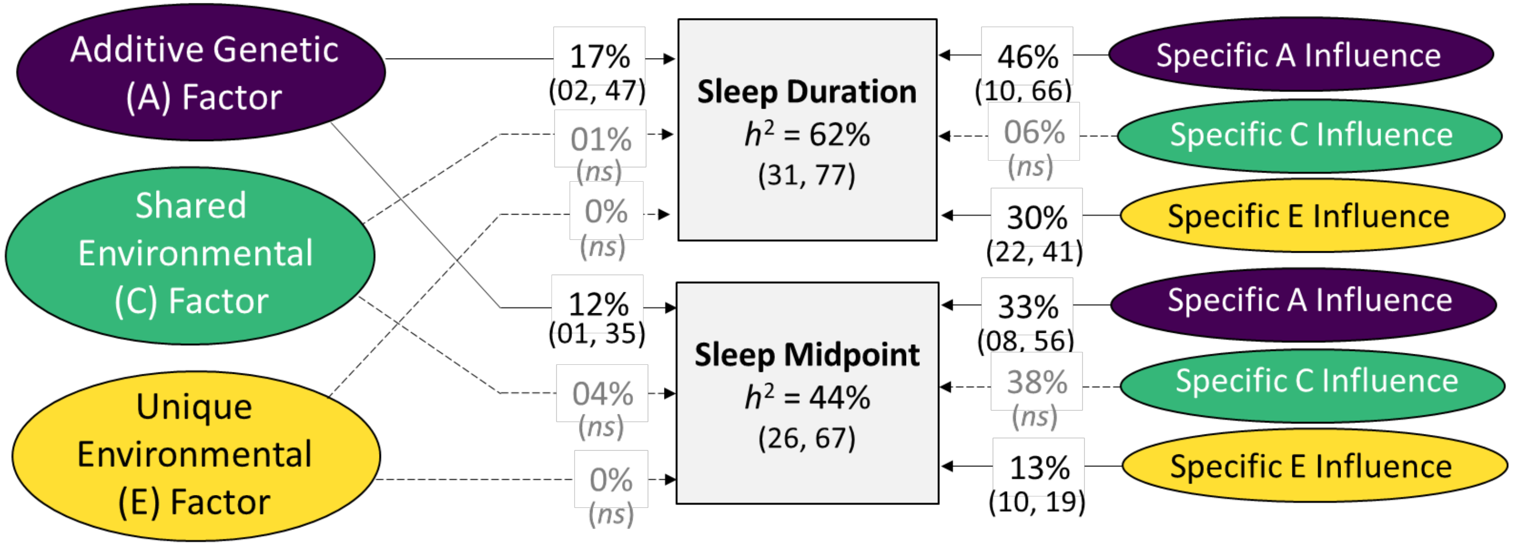

Figure S2. Conceptual model representations derived from Cholesky decompositions between sleep duration and sleep midpoint. Estimates are derived from two bivariate Cholesky decompositions as described in Figure S1 c; specific estimates tend to be larger in a Cholesky decomposition than in an independent pathway model, see Loehlin (1996) [53], still, this model agrees that the association is entirely due to a common genetic source i.e. 37% (17/46) of the genetic variance in sleep duration and 37% (12/33) of the genetic variance in midpoint overlapped. h^2 = heritability estimate; dashed lines represent non-significant pathways.

Figure S3

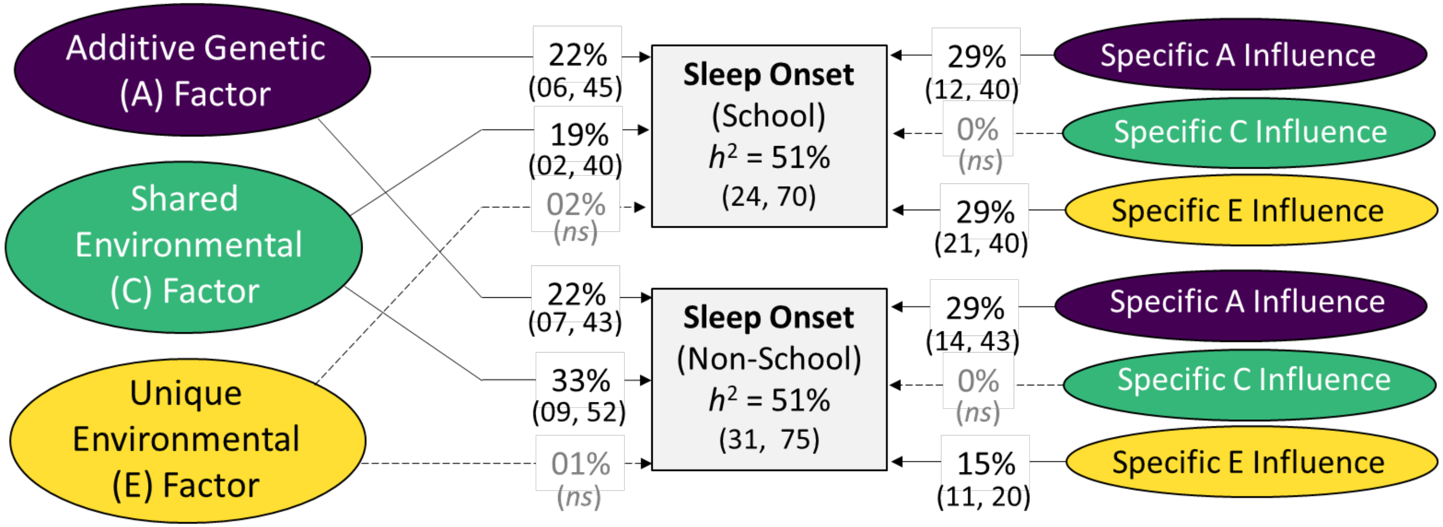

Additive Genetic (A) Factor
Shared Environmental (C) Factor
Unique Environmental (E) Factor
22% (06, 45)
19% (02, 40)
02% (ns)
22% (07, 43)
33% (09, 52)
01% (ns)
Sleep Onset (School) h^2 = 51% (24, 70)
Sleep Onset (Non-School) h^2 = 51% (31, 75)
29% (12, 40)
0% (ns)
29% (21, 40)
29% (14, 43)
0% (ns)
15% (11, 20)
Specific A Influence
Specific C Influence
Specific E Influence
Specific A Influence
Specific C Influence
Specific E Influence

a.

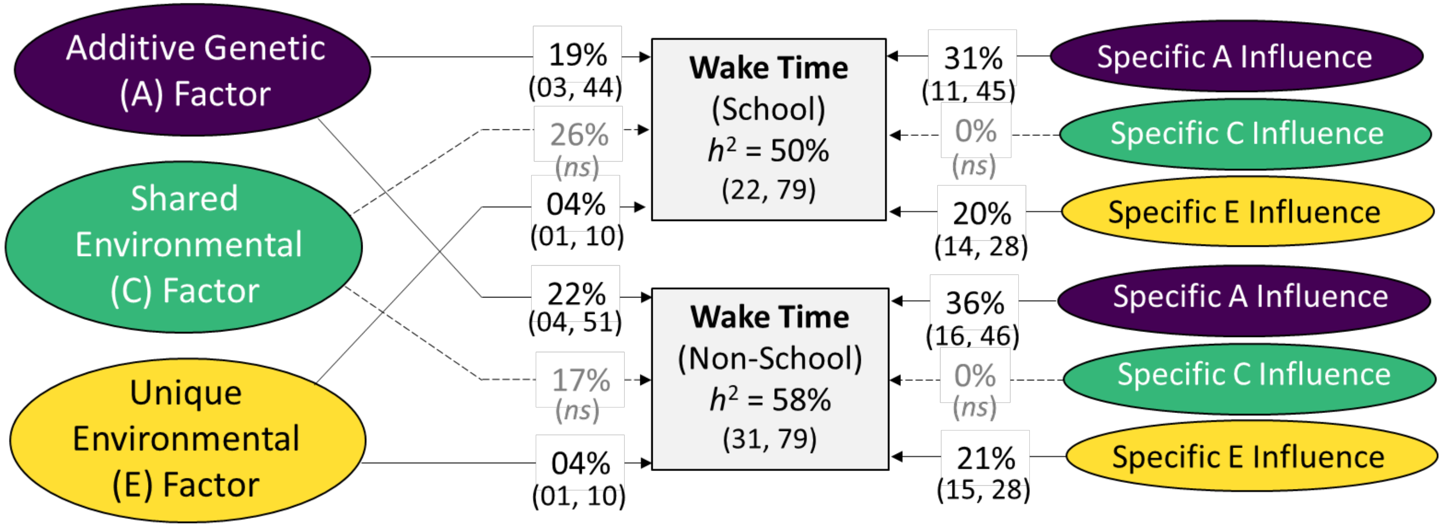

Additive Genetic (A) Factor
Shared Environmental (C) Factor
Unique Environmental (E) Factor
19% (03, 44)
26% (ns)
04% (01, 10)
22% (04, 51)
17% (ns)
04% (01, 10)
Wake Time (School) h^2 = 50% (22, 79)
Wake Time (Non-School) h^2 = 58% (31, 79)
31% (11, 45)
0% (ns)
20% (14, 28)
36% (16, 46)
0% (ns)
21% (15, 28)
Specific A Influence
Specific C Influence
Specific E Influence
Specific A Influence
Specific C Influence
Specific E Influence

b.

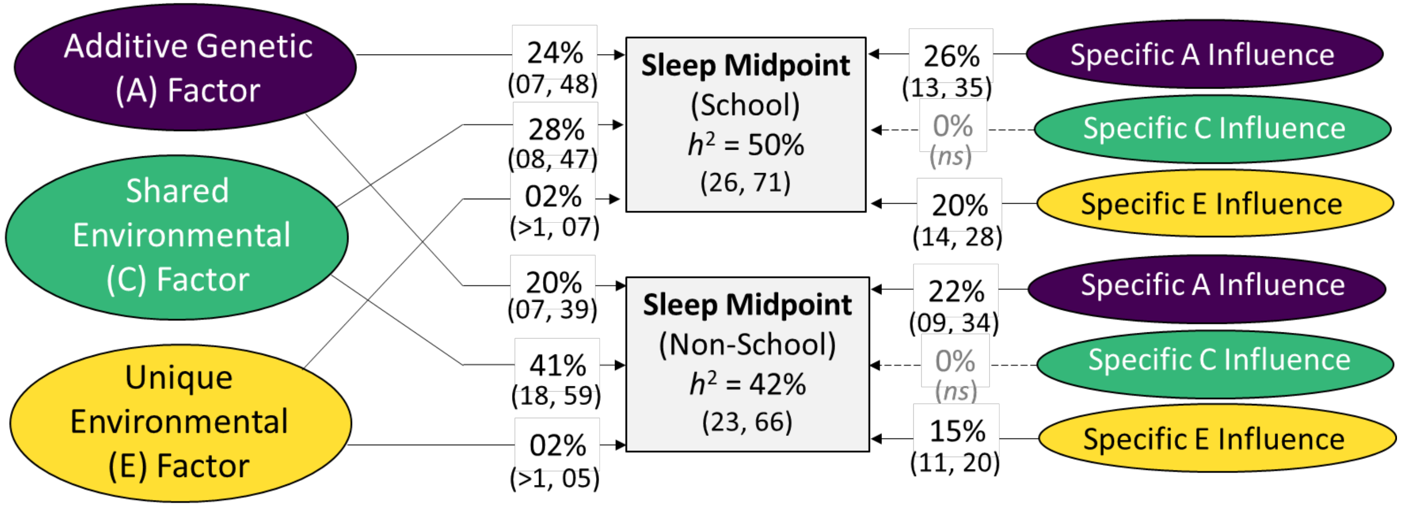
Additive Genetic (A) Factor
Shared Environmental (C) Factor
Unique Environmental (E) Factor
24% (07, 48)
28% (08, 47)
02% (>1, 07)
20% (07, 39)
41% (18, 59)
02% (>1, 05)
Sleep Midpoint (School) h² = 50% (26, 71)
Sleep Midpoint (Non-School) h² = 42% (23, 66)
26% (13, 35)
0% (ns)
20% (14, 28)
22% (09, 34)
0% (ns)
15% (11, 20)
Specific A Influence
Specific C Influence
Specific E Influence
Specific A Influence
Specific C Influence
Specific E Influence

c.

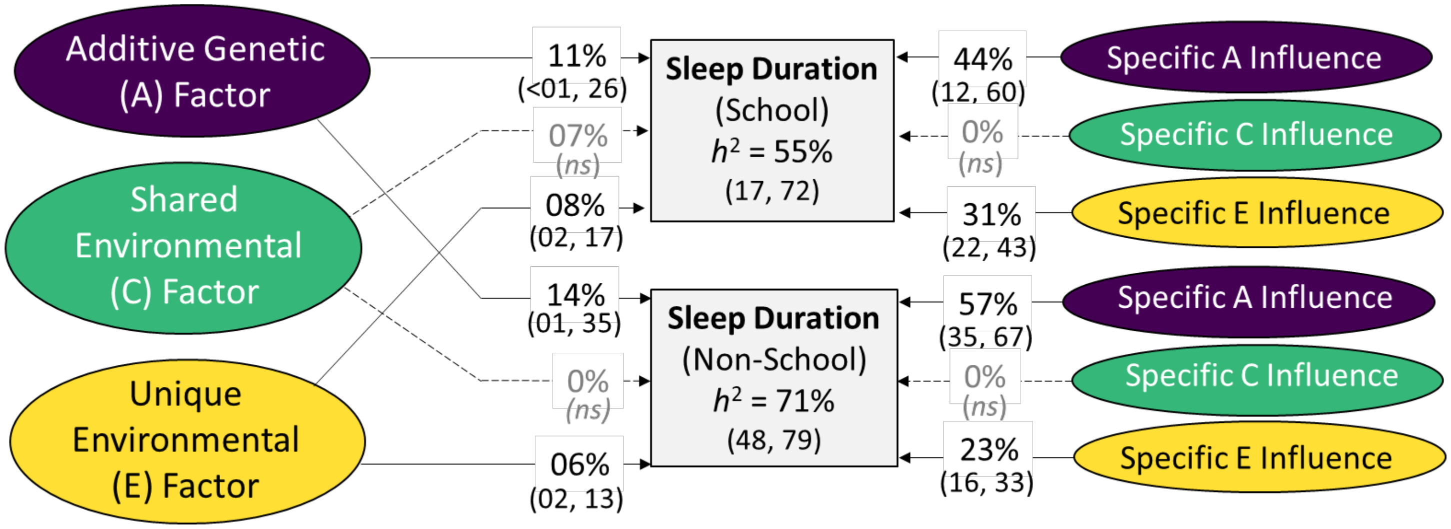
Additive Genetic (A) Factor
Shared Environmental (C) Factor
Unique Environmental (E) Factor
11% (<01, 26)
07% (ns)
08% (02, 17)
14% (01, 35)
0% (ns)
06% (02, 13)
Sleep Duration (School) h² = 55% (17, 72)
Sleep Duration (Non-School) h² = 71% (48, 79)
44% (12, 60)
0% (ns)
31% (22, 43)
57% (35, 67)
0% (ns)
23% (16, 33)
Specific A Influence
Specific C Influence
Specific E Influence
Specific A Influence
Specific C Influence
Specific E Influence

d.

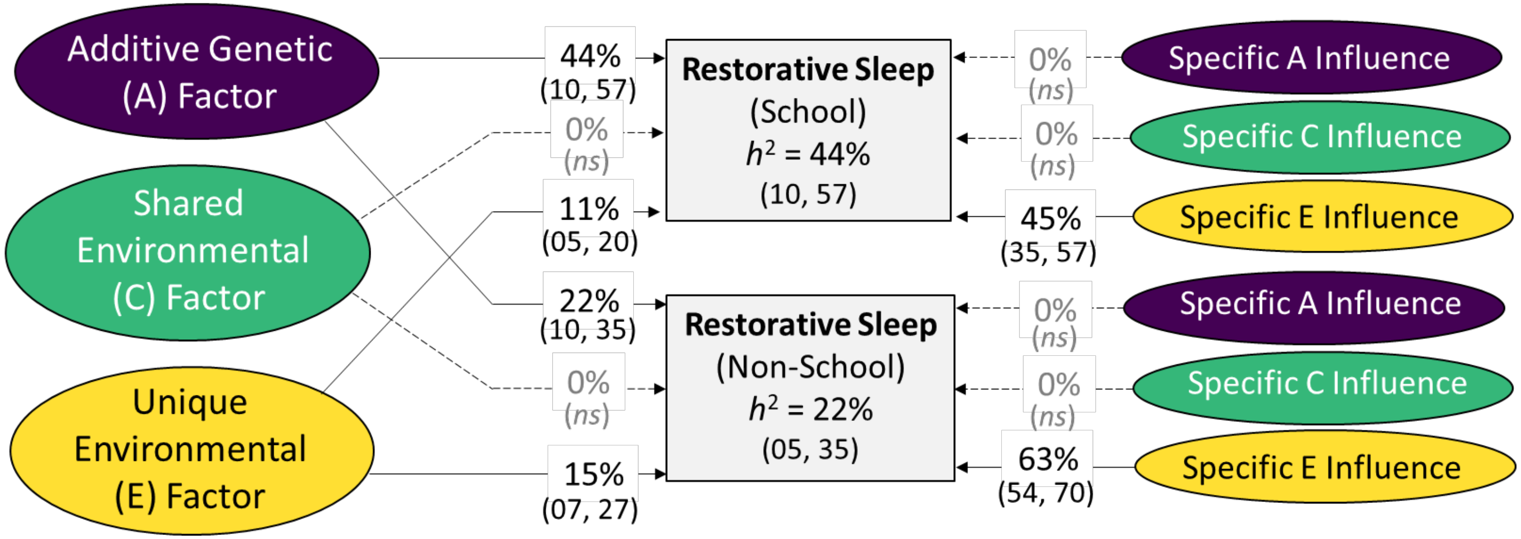

e.

Figure S3 a-e. Conceptual model representations derived from Cholesky decompositions between school and non-school nights for **a)** sleep onset, **b)** wake time, **c)** sleep midpoint, **d)** sleep duration, and **e)** restorative sleep. These Cholesky decompositions agree that for all accelerometry measures both common (left-hand circles) and specific (right-hand circles) genetic sources influence school and non-school nights in adolescence, with 34 to 71% heritability. In contrast, variance due to shared (family) environment completely overlapped, with no evidence of a shared environmental influence specific to night type. For restorative sleep, genetic variance for school and non-school nights completely overlapped, and there was no genetic variance specific to night type, and no evidence of any variance due to shared (family) environment. h^2 = heritability estimate; dashed lines represent non-significant pathways.
